# Field Validation of Food Service Listings: A Comparison of Commercial and Online Geographic Information System Databases

**DOI:** 10.3390/ijerph9082601

**Published:** 2012-07-25

**Authors:** Laura Seliske, William Pickett, Rebecca Bates, Ian Janssen

**Affiliations:** 1 Department of Community Health & Epidemiology, Queen’s University, Kingston, ON K7L 3N6, Canada; Email: lseliske@gmail.com (L.S.); will.pickett@queensu.ca (W.P.); 2 Clinical Research Center, Angada 3, Kingston General Hospital, 76 Stuart St., Kingston, ON K7L 2V7, Canada; 3 School of Kinesiology & Health Studies, Queen’s University, 28 Division Street, Kingston, ON K7L 3N6, Canada; Email: rcbates@lakeheadu.ca

**Keywords:** built environment, food service place databases, field validation

## Abstract

Many studies examining the food retail environment rely on geographic information system (GIS) databases for location information. The purpose of this study was to validate information provided by two GIS databases, comparing the positional accuracy of food service places within a 1 km circular buffer surrounding 34 schools in Ontario, Canada. A commercial database (InfoCanada) and an online database (Yellow Pages) provided the addresses of food service places. Actual locations were measured using a global positioning system (GPS) device. The InfoCanada and Yellow Pages GIS databases provided the locations for 973 and 675 food service places, respectively. Overall, 749 (77.1%) and 595 (88.2%) of these were located in the field. The online database had a higher proportion of food service places found in the field. The GIS locations of 25% of the food service places were located within approximately 15 m of their actual location, 50% were within 25 m, and 75% were within 50 m. This validation study provided a detailed assessment of errors in the measurement of the location of food service places in the two databases. The location information was more accurate for the online database, however, when matching criteria were more conservative, there were no observed differences in error between the databases.

## 1. Introduction

The built environment in which people live can have a major influence on obesity and its behavioral determinants, physical activity and diet [1]. Several studies have documented relationships between the availability of food service places in the local environment (e.g., fast food restaurants, convenience stores) and eating behaviors and obesity [2,3,4,5]. Most studies rely on geographical information systems (GIS) databases to measure the food service place listing. Quantification of positional error in GIS databases is important because it accounts for some of the measurement bias present in etiological studies of the food environment.

To date, seven validation studies have examined the accuracy of the information on food service place locations provided by GIS databases [6,7,8,9,10,11,12]. Many of the existing studies classified food service places as present or absent at their listed address, rather than measuring distances between the true and reported locations. This approach does not provide information on whether the true location of the food service place is a few meters or several meters away from the listed location. This has important implications for whether people can access the food service places by walking. Furthermore, existing studies have had small sample sizes (n < 200) [11] and occurred within a single city [7,9,11], which may limit the applicability of their findings to other locations or to non-urban areas. Our study objective was to evaluate the positional accuracy of the geocoded addresses of food service places provided by two GIS databases in urban and non-urban areas.

## 2. Experimental Section

### 2.1. Sampling Approach

We measured the food service places surrounding 34 schools. Schools were chosen as the sampling unit because this study was part of a larger research program examining the food environment around schools and how it relates to students’ eating behaviours. The schools were located in 22 cities and towns across southern Ontario, Canada. Nine schools were located in non-urban areas (<10,000 people) and 25 were located within urban areas (>10,000 people) [13]. A 1 km circular buffer was created around each school using ArcGIS (ESRI, version 9.3, Redlands, CA, USA) and no buffers overlapped. The location of various types of food service places was obtained from two databases and geocoded within a 1 km circular buffer surrounding each school. Their locations were then confirmed by conducting a field validation.

### 2.2. Food Service Places

The locations of the food service places were obtained from a commercial database (InfoCanada) and an online Yellow Pages database [14] in March through May of 2010. The North American Industry Classification System was used to obtain multiple categories of food service places from the InfoCanada database, including: full-service restaurants, limited-service restaurants, snack and non-alcoholic beverage bars, and convenience stores. These food service places were chosen because it was expected that students would purchase food from them, rather than from grocery stores or supermarkets. We merged the snack and non-alcoholic beverage bars into the limited-service restaurant category to maintain consistency of categories across the databases. For the Yellow Pages database, full-service restaurants and convenience stores were obtained with the keywords “restaurant” and “convenience store”, respectively. Limited-service restaurants were obtained with the keywords “ice-cream & frozen desserts”, “sandwiches”, and “donut-retail”. In addition, chain limited service restaurants which appeared in the full-service search results were re-categorized as limited-service restaurants (available from the authors upon request).

The address of each food service place was geocoded using the North American Address Locator in ArcGIS. For geocoded locations which received a match score of less than 80 out of 100, additional information was sought to improve the score to 80 or higher. If that was not possible, x,y coordinates were obtained after visual inspection of the location using the Street View tool in Google Earth [15].

The actual location of the food service places was obtained in the field study in June through August of 2010. Each food service place was searched for in the field, and if it was not initially found, a phone call was made to ensure it existed and to help locate its position. Food service places were considered to exist if components of the name provided by the databases corresponded to the food service place found in the field. The location of each food service place was recorded at the curb side street entrances using a Garmin Dakota 10 handheld Global Positioning System (GPS) device (Garmin International Inc., Olathe, KS, USA) to record a waypoint containing its geographic coordinates. In downtown areas where there were no distinct curb side street entrances, the position of the storefront entrance was measured instead. To help ensure a stable reading, the waypoint provided by the GPS unit was monitored until it stabilized, after which the waypoint was recorded. 

### 2.3. Statistical Analysis

Differences in the GIS- and GPS-derived locations were determined by measuring the Euclidian (straight line) distance in ArcGIS. Because values for these distances were skewed, medians were reported and the Wilcoxon rank-sum test was used to determine if the distances differed between the GIS databases. We also determined the proportion of the food service place addresses which were located within the 1 km buffer, and also within 100 m, 50 m, and 25 m of the true GPS-measured location. Chi-square and Fisher’s exact tests were used to determine whether the proportion of GIS-measured food service places located within these distances differed between the two databases.

## 3. Results and Discussion

### 3.1. Results

The InfoCanada and Yellow Pages GIS databases provided the locations for 973 and 675 food service places, respectively, in the 1 km buffer surrounding the 34 schools. Overall, 749 (77.1%) and 595 (88.1%) of these were located within the field, respectively. For urban schools, the proportion of all categories of food service places found within the 1 km buffer was higher for the Yellow Pages database, with the exception of convenience stores (Table 1). The proportion of the listed food service places found within a specific distance decreased as the size of the distance got smaller (Table 1). For example, for urban schools, the proportion of limited-service restaurants in the Yellow Pages database that were within 100 m of their true location was 77%; 53% were within 50 m and only 26% were within 25 m.

**Table 1:** The proportion of food service places in the GIS databases that were found in the field validation.

Distance		Urban			
		InfoCanada		Yellow Pages	
		N	% (95% CI)	N	% (95% CI)
**Within 1 km Buffer**					
****	All Food Service Places	624	76 (73–79)	523	88 (85–91) ^†^
	Full Service	283	72 (67–77)	320	88 (85–92) ^†^
	Limited Service	269	80 (75–85)	124	88 (82–94) *
	Convenience	72	77 (68–87)	79	86 (78–94)
**Within 100 m**					
	All Food Service Places	558	68 (64–72)	473	80 (76–83) ^†^
	Full Service	261	67 (61–72)	294	81 (77–86) ^†^
	Limited Service	231	69 (63–75)	109	77 (69–85)
****	Convenience	66	71 (60–82)	70	76 (66–86)
**Within 50 m**					
	All Food Service Places	449	55 (50–59)	382	64 (59–69) ^†^
	Full Service	229	58 (52–69)	250	69 (63–75) ^†^
	Limited Service	166	49 (42–57)	74	53 (41–64)
****	Convenience	54	58 (45–71)	58	63 (51–75)
**Within 25 m**					
	All Food Service Places	297	36 (31–42)	245	41 (35–47)
	Full Service	164	42 (34–49)	168	46 (39–54)
	Limited Service	97	29 (20–38)	36	26 (11–40)
	Convenience	36	39 (23–55)	41	45 (29–60)
**1 km Buffer**					
	All Food Service Places	125	83 (77–90)	72	90 (83–97)
	Full Service	55	75 (64–87)	48	91 (82–99) *
	Limited Service	57	92 (85–99)	16	89 (74–100)
	Convenience	13	87 (68–100)	8	89 (67–100)
**100 m**					
	All Food Service Places	114	76 (68–84)	66	83 (73–92)
	Full Service	50	69 (56–81)	45	85 (74–95) *
	Limited Service	51	82 (72–93)	14	78 (56–99)
	Convenience	13	87 (68–100)	7	78 (47–100)
**50 m**					
	All Food Service Places	103	69 (60–78)	57	71 (60–83)
	Full Service	47	64 (51–78)	41	77 (65–90)
	Limited Service	43	69 (56–83)	10	56 (25–87)
****	Convenience	13	87 (68–100)	6	67 (29–100)
**25 m**					
	All Food Service Places	81	54 (43–65)	50	63 (49–76)
	Full Service	38	52 (36–68)	37	70 (55–85) *
	Limited Service	33	53 (36–70)	9	50 (17–83)
	Convenience	10	67 (38–96)	4	44 (0–93)

^†^ = proportion of food service places differs between sources at a *p* value ≤ 0.01;* = proportion of food service places differs between sources at a *p* value ≤ 0.05.

Table 2 provides the median positional error, defined as the distance between the listed and true food service place locations. The positional error did not differ between InfoCanada (24.6 m, interquartile range: 13.2–51.0 m) and the Yellow Pages (25.6 m, interquartile range: 13.1–51.7 m) databases.

**Table 2:** Positional error (meters) of food service place locations provided the GIS databases

		InfoCanada		Yellow Pages		P value
		N	Median (IQR)	N	Median (IQR)	
**Urban Schools**						
	All Food Service	628	26.9 (13.5–54.4)	525	27.7 (13.8–51.7)	0.98
	Places Full Service	283	20.9 (12.3–41.9)	320	22.5 (12.2–44.2)	0.34
	Limited Service	272	37.6 (16.8–67.4)	125	44.0 (21.1–69.4)	0.31
	Convenience	7	24.6 (13.5–49.9)	80	24.6 (13.7–60.6)	0.61
**Non–Urban Schools**						
	All Food Service Places	121	16.8 (10.3–30.3)	70	14.4 (8.1–27.6)	0.20
	Full Service	55	16.7 (9.7–30.3)	48	14.4 (7.6–23.4)	0.70
	Limited Service	54	17.1 (10.4–35.1)	15	17.0 (9.5–54.3)	0.89
	Convenience	12	16.8 (13.9–23.6)	7	13.9 (7.4–32.8)	0.37

IQR = Interquartile Range.

### 3.2. Discussion

The key findings for this study were that the Yellow Pages directory provided a greater proportion of the listed food service places in the 1 km buffer, but the positional error did not differ between GIS databases. When considering the presence or absence of food service places within a 1 km buffer, approximately 75% or more of the listed food service places were found in the field. However, when more precise thresholds were considered (e.g., within 25 m), less than half of the food service places were found in the field.

The percentage of food service places located within the 1 km buffer was comparable to results found by other studies. For example, Hosler and Dharssi [8] were able to locate 81.7% of the listed food service places provided by government sources in Albany, New York. Lake *et al*. [9] assessed the information provided by two online sources (Yellow Pages and Yell.com) in Newcastle-Upon-Tyne, England. They located 82.4% and 79.1% food service places, respectively. Liese *et al*. [10] assessed the validity of food service place databases in urban and rural locations in South Carolina and were able to find 77.7% and 86.5% of the food service places listed by the commercial sources of Dun & Bradstreet and InfoUSA, respectively. Similarly, Sharkey and Horel [12] found that a similar proportion of food service places listed in publicly available databases were not found in the field (18.9%) in rural Texas. 

When comparing the proportion of food service places in the online and commercial GIS databases, we found a higher proportion of those listed in the online database. This corresponded to the findings of Paquet *et al*. [11], who found that a combined source of several online databases had a greater proportion of food service places found in the field (98%) compared to a commercial source (90%). The higher validity of the online sources may be explained by how frequently the databases are updated. The location information for InfoCanada is valid for 6 months, while the Yellow Pages provides monthly subscriptions. 

Few studies have measured the positional accuracy of food service place databases. Liese *et al*. [10] found that approximately half of the food service places provided by commercial sources (Dun & Bradstreet and InfoUSA) were within 100 m of their true locations and this varied by urban-rural status. Our results had a greater percentage of food service places found within 100 m of the listed locations for both GIS databases and there were no differences between urban and non-urban schools, although this may be due a small sample size for the non-urban schools in our study. 

There are some limitations to our study that warrant consideration. Because we were primarily interested in determining whether the geocoded address of a food service place was in close proximity to its actual address, we did not assess whether the listed address was correct. Thus, some of the positional error may be due to incorrect address information being listed in the databases. Also, due to the large number of listed food service places in this study, it was not feasible to measure the presence of food service places located within the 1 km buffer that did not appear in the GIS databases. Thus, we were unable to calculate the sensitivity of the databases. The category of the food service places (e.g., chain or non-chain) not found in the field was not collected. Also, we did not assess whether the categorization of food service places was correct, which may have introduced some misclassification between food service place types. In addition, there were small numbers of food service places in non-urban locations, which may account for the lack of statistically significant findings in those areas. 

With respect to the GPS measures, we were unable to calculate the dilution of precision, which assesses the accuracy of the GPS readings. Some of the measurement error for both databases may be explained by the fact that GIS software estimates street address locations by uniformly distributing street address numbers along road segments. These estimated locations may not precisely match the actual street address locations.

## 4. Conclusions

Half of the food service places were positioned within approximately 25 m of their true location by the two GIS databases, and 75% were positioned within approximately 50 m. The Yellow Pages database provided a higher proportion of matches within the 1 km buffer compared to the InfoCanada database. 
